# Lumbosacral Transitional Vertebrae in Adolescents: Effects on the Short-Term Outcomes of Percutaneous Endoscopic Lumbar Discectomy

**DOI:** 10.1155/2021/9911579

**Published:** 2021-07-12

**Authors:** Yong Huang, Lu Mao, Hang Shi, Guanrui Ren, Lei Zhu, Rui Zhang, Zhengming Shan, Xiaotao Wu

**Affiliations:** ^1^School of Medicine, Southeast University, Nanjing, China; ^2^The Department of Orthopedics, Zhongda Hospital of Southeast University, Nanjing, China

## Abstract

**Objective:**

To investigate whether lumbosacral transitional vertebrae (LSTV) affects the clinical outcomes of percutaneous endoscopic lumbar discectomy (PELD) in adolescent patients with lumbar disc herniation (LDH).

**Methods:**

This was a retrospective study with two groups. Group A was made up of 22 adolescent LDH patients with LSTV (18 males and 4 females). Group B was made up of 44 adolescent LDH patients without LSTV (36 males and 4 females), who were matched to group A for age, sex, and body mass index. All patients underwent PELD at the L4/5 or L5/S1 single level and were followed up at 18 months after surgery. We identified LSTV on radiographs and computed tomography and assessed the imaging characteristics of all patients. Outcomes were evaluated through a numerical rating scale (NRS), the Oswestry Disability Index (ODI), the modified MacNab grading system, and the incidence of additional lumbar surgery.

**Results:**

At 18 months after PELD, both groups had significant improvements in the mean NRS scores of low back pain (LBP) or leg pain and the ODI scores. In terms of the MacNab criteria, 90.9% in group A and 93.2% in group B showed excellent or good outcomes. The mean NRS scores of LBP or leg pain, ODI score, and MacNab grade after surgery were not significantly different between the 2 groups. Two patients (one patient had a recurrence; one patient had a new lumbar disc herniation) in group A and 3 patients (one patient had a recurrence; two patients had new lumbar disc herniations) in group B underwent additional lumbar surgery.

**Conclusions:**

Our study suggests that in terms of pain relief, life function improvement, and the incidence of additional lumbar surgery, LSTV has no effect on the short-term clinical outcomes of PELD in adolescents. A new lumbar disc herniation is an important reason for additional surgery in adolescents, regardless of the LSTV status.

## 1. Introduction

Most adolescent patients with lumbar disc herniation (LDH) can be cured by conservative treatment [1, 2]. Adolescent patients with symptoms refractory to conservative treatment for at least 4 ~ 6 weeks or symptoms exceeding the patient's tolerance may consider surgical procedures [3–5]. Previous studies found that percutaneous endoscopic lumbar discectomy (PELD) is a safe and effective procedure for the treatment of LDH in adolescents [3, 4, 6, 7]. However, some adolescent patients have recurrent back pain, leg pain, or additional disc herniations [8, 9]. In clinical work, we found many adolescent patients with LDH accompanied by lumbosacral transitional vertebra (LSTV). We speculated that LSTV might be an important factor for poor outcomes of PELD in adolescents.

LSTV is a common anatomical variant defined as the transverse processes of the last lumbar vertebra fused with the sacrum. In the general population, LSTV has a prevalence of 7% to 36% [10–14]. Tang et al. reported that the prevalence of LSTV is 15.8% in the Chinese Han population [15]. Previous studies have found that specific LSTV types, namely, types II, III, and IV, are significantly associated with low back pain (LBP) [10, 16–20]. LSTV leads to relative spinal hypermobility and disc degeneration progresses at the cephalad segment in adolescents [10, 11, 19, 21, 22]. Do adolescent patients with LSTV have poorer outcomes after PELD than those without LSTV?.

We used a retrospective study to investigate whether the presence of LSTV affects the clinical outcomes of PELD in adolescent patients. To our knowledge, few studies have analyzed the effect of LSTV on the outcomes of discectomy surgery.

## 2. Materials and Methods

### 2.1. Study Population

This research was approved by the Institutional Review Board at the Zhongda Hospital affiliated with Southeast University. One hundred thirty-four adolescent patients with lumbar disc herniation who underwent PELD were enrolled in this study between January 2014 and June 2019. The inclusion criteria were as follows: (1) all patients between 12 and 21 years old who received PELD treatment at L4/5 or L5/S1 single level; (2) their indications for surgical treatment were symptoms refractory to conservative treatment for at least 6 weeks or symptoms exceeding the patient's tolerance; (3) these adolescent patients were diagnosed with LDH based on their symptoms and physical examinations, and the diagnoses were confirmed by lumbar spine radiographs, two-dimensional computed tomography (CT), and magnetic resonance imaging (MRI); (4) all surgical procedures were performed by two experienced spine surgeons; and (5) the follow-up time was at 18 months after the operation. The exclusion criteria included the following: loss to follow-up, spine malformation, previous lumbar spine surgery, and surgery for L3/4 level LDH or L4/5 and L5/S1 two levels.

**Figure 1:**
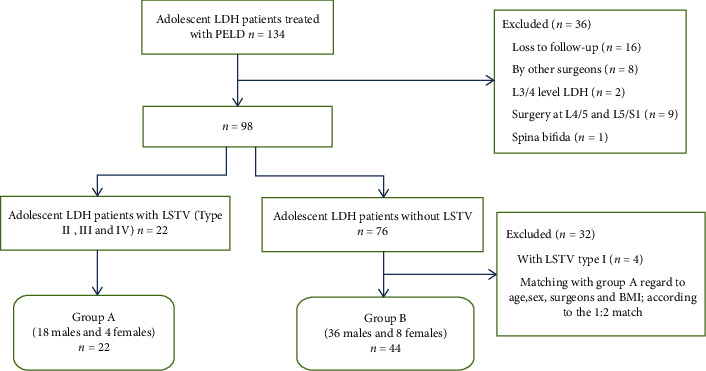
Flowchart of patients in the study. BMI: body mass index; LDH: lumbar disc herniation; LSTV: lumbosacral transitional vertebrae; PELD: percutaneous endoscopic lumbar discectomy.

Thirty-six patients were excluded, and the remaining 98 patients were divided into two groups. Group A was made up of patients with LSTV (types II, III, and IV) including 18 males and 4 females. Castellvi et al. reported that type I represents a “forme fruste” of the lumbosacral transitional vertebra and shows no difference in the incidence of the location of herniations [10]. Therefore, we defined LSTV as Castellvi type II, III, or IV in this study. Forty-four patients without LSTV including 36 males and 8 females (age, sex, and BMI matched with group A) were selected to form group B as the control group (Figure 1).

### 2.2. Imaging Evaluation

The CT scans were performed using a 64-channel system (SOMATOM). The MRI scans were performed using a 1.5 T MRI system (Signa Excite scanner). All radiographs were reviewed on a picture archiving and communication system (Neusoft PACS viewer) by 2 independent observers. If their interpretations were different, we consulted a third observer.

#### 2.2.1. Image Identification of LSTV

**Figure 2:**
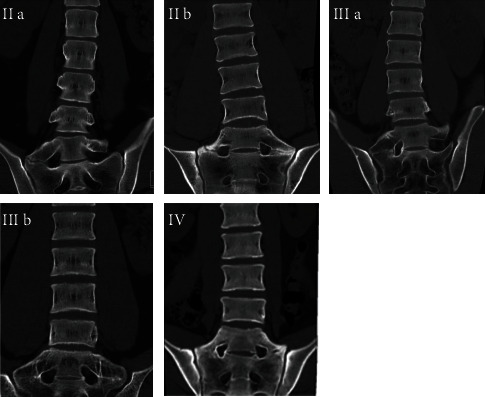
Identification of LSTV. Typical images of Castellvi type IIa, IIb, IIIa, IIIb, and IV are shown on coronal reconstructed two-dimensional computed tomography.

We identified LSTV and the Castellvi classification by counting down from the last thoracic vertebra on the anteroposterior radiograph of the lumbosacral vertebrae. Then, we used CT to reidentify the Castellvi classification of LSTV: type I, enlarged transverse processes without pseudoarticulation or fusion with the sacral bone; type II, enlarged transverse process with pseudoarticulation with the sacral bone (a, unilateral; b, bilateral); type III, enlarged transverse processes with fusion with the sacral bone (a, unilateral; b, bilateral); and type IV, enlarged transverse processes with pseudoarticulation and fusion with the sacral bone [10]. We defined LSTV as Castellvi type II, III, or IV classification in this study (Figure 2).

#### 2.2.2. Disc Herniation

We identified the segments of the herniated disc on sagittal MRI. The herniation type was categorized as central, centrolateral, foraminal, or far lateral on axial MRI.

#### 2.2.3. Sacral Slope Angle (SSA)

**Figure 3:**
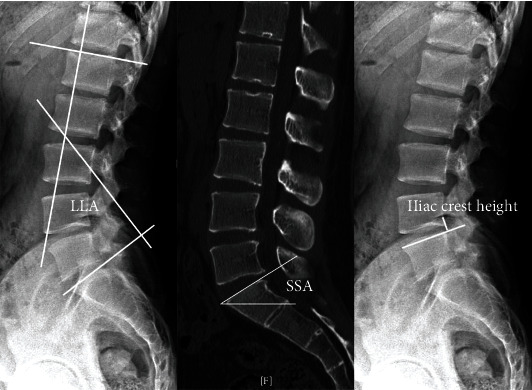
Measurement of LLA, SSA, and iliac crest height. SSA: sacral slope angle; LLA: lumbar lordosis angle; iliac crest height (we measured the distance on lateral radiographs between the upper margin of the L5 vertebrae and the highest point of the iliac crest as the iliac crest height).

SSA was measured in the midsagittal CT plane and defined as the angle formed by the line of the upper end plate of the sacrum and the horizon (Figure 3).

#### 2.2.4. Lumbar Lordosis Angle (LLA)

LLA was measured in the sagittal plane between the lines of the upper endplate of L1 and S1 (adapted to the sagittal plane) (Figure 3).

#### 2.2.5. Iliac Crest Height

We measured the distance on lateral radiographs between the upper margin of the L5 vertebrae and the highest point of the iliac crest as the iliac crest height (Figure 3).

### 2.3. Surgical Procedure

PELD was performed under local anesthesia by two experienced spine surgeons using transforaminal procedures. The patients were placed prone on a radiolucent orthopedic surgery bed. The marker line was verified by anteroposterior C-arm view (the midpoint of the inferior vertebral endplate and the apex of the superior articular process) and side view (posterior margin of the lumbar vertebra). A piercing point was verified 8–12 cm lateral to the posterior midline. The puncture needle was inserted gently, with the beveled opening pointing to the traversing root. After passing through a multistage expander along the needle, the working casing and endoscope were inserted. If narrowing of the foramen impeded the surgical pathway, a trephine was used for foraminotomy; this procedure also helps improve the surgical field. Herniated disc fragments causing root impingement were removed by straight/angulated forceps. We evaluated the extent of release by gently pulling the nerve root. Radiofrequency ablation was used to ablate the blood vessels and shape the fiber ring and posterior longitudinal ligament. Patients were allowed to walk with a lumbar brace 8-12 hours postoperatively. They were advised to wear a lumbar brace for 1 month and to avoid strenuous activity for 3 months after surgery.

### 2.4. Clinical Outcome Evaluation

A numerical rating scale (NRS) was used for the assessment of LBP or leg pain intensity, and the Oswestry Disability Index (ODI) was used for the assessment of functionality preoperatively. The patients were followed up with the same questionnaire at 18 months after surgery. In addition, the modified MacNab grading system was used to determine the effectiveness of treatment at 18 months after surgery (patients who underwent additional lumbar spine surgery were classified as poor). We also recorded the incidence of additional surgery within 18 months after PELD. Preoperative assessments were performed in person, and postoperative assessments were conducted by telephone at 18 months after surgery.

### 2.5. Statistical Analysis

We used SPSS version 25.0 software (IBM Corp., Armonk, New York, USA) for statistical analysis. Pearson's chi-square test or Fisher's exact test was used for the categorical data. Student's *t*-test was used to confirm intergroup differences in cases with normal distribution, whereas the Mann–Whitney *U* test was used to compare variables between the 2 groups with nonnormal distributions. *p* < 0.05 indicates significance.

## 3. Results

### 3.1. Demographic Characteristics

Twenty-two patients in group A (with LSTV) and 44 patients in group B (without LSTV) were reviewed. A comparison of the demographic and baseline clinical characteristics of the 2 groups is shown in Table 1. There was no significant difference between the 2 groups in age (*p* = 0.391), sex (*p* = 1.000), body mass index (*p* = 0.641), surgeon (*p* = 0.544), duration of symptoms (*p* = 0.428), history of injury (*p* = 0.498), clinical symptoms (*p* = 0.150), and positive straight leg raise test (*p* = 0.888) (Table 1).

### 3.2. Imaging Evaluation

We defined LSTV as Castellvi type II, III, or IV. There were 11 type IIa, 6 type IIb, 2 type IIIa, 2 type IIIb, and 2 type IV in group A. Typical images are shown in Figure 2.

A comparison of the imaging characteristics of the two groups is shown in Table 2. Patients with LSTV had a larger SSA and a higher iliac crest (*p* < 0.001 and <0.001). However, no difference was detected in operated level (*p* = 0.473), type of herniation (*p* = 0.702), calcification (*p* = 1.000) and LLA (*p* = 0.395) between the 2 groups. Herniation levels on MRI were in various formats, and many patients (22.7% in group A; 43.2% in group B) had multisegmental lumbar disc herniation (Table 2).

### 3.3. Clinical Outcome Evaluation

There was no significant difference in the preoperative NRS scores of LBP or leg pain between the 2 groups (*p* = 0.081 and 0.156). According to the NRS scores, both groups had significant improvements in pain relief at 18 months after surgery. The NRS scores of LBP or leg pain were not significantly different between the 2 groups postoperatively (*p* = 0.764 and 0.213) (Table 3).

There was no significant difference in preoperative ODI scores between the 2 groups (*p* = 0.874). The ODI scores significantly improved at 18 months after surgery in both groups. When the short-term outcomes were compared, the ODI scores were not significantly different between the 2 groups (*p* = 0.117) (Table 3).

According to the results of the modified MacNab grading system, 90.9% in group A and 93.2% in Group B showed excellent or good outcomes, and there was not significant difference between the 2 groups at 18 months after surgery (*p* = 1.000) (Table 3).

**Figure 4:**
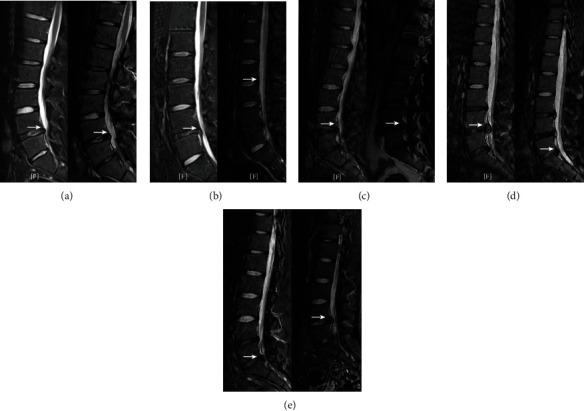
MRI before the first surgery and the additional surgery (the white arrows indicate lumbar disc herniation). One patient in group A exhibited a recurrence (a). One patient in group A presented a new lumbar disc herniation (b). One patient in group B exhibited a recurrence (c). Two patients in group B presented new lumbar disc herniations (d and e).

There were no major complications including neurovascular injury, CSF leakage, or infection in either group. At 18 months after surgery, the number of simple LBP was greater than the number with pain in the back and leg (in group A, 8 vs. 4; in group B, 13 vs. 6). The simple LBP was usually short and mild, which was often caused by excessive sitting and was relieved by conservative treatment. Leg pain had a strong impact on the degree of pain and life function. Four patients in group A and 6 patients in group B had pain in the back and leg, and half of each group was relieved by conservative treatment, but the remaining patients underwent additional surgery which resulted in good recovery at the follow-up time (one patient in group A exhibited a recurrence at 12 months after surgery, and another experienced a new lumbar disc herniation at 9 months; in group B, one patient exhibited a recurrence at 10 months, and two experienced new lumbar disc herniations at 3 months and 12 months) (Figure 4). In this study, the same diagnosis and on the same level and laterality was considered a recurrence, while a new segmental disc herniation was considered a new lumbar disc herniation. There was no significant difference between the 2 groups in the incidence of additional lumbar surgery (*p* = 1.000) (Table 3).

## 4. Discussion

LSTV protects the disc at the transitional level and predisposes the adjacent cephalad segment to greater mobility [10, 11, 19, 21, 22]. LSTV is positively correlated with the prevalence and severity of LBP [10, 11, 16, 19, 20]. Zhang et al. reported that LSTV is associated with LDH and that sacralization of L5 may contribute to L4/5 disc herniation in adolescent patients through a retrospective case-control analysis [21]. Bertolotti's syndrome refers to the association of back pain with lumbosacral transitional vertebrae [11]. Quinlan et al. reported that Bertolotti's syndrome was present in 11.4% (20 patients) of the under-30 age group [19]. We hypothesized that LSTV might affect the postoperative outcome of PELD in adolescents. In particular, LSTV might lead to poor LBP relief after PELD.

In our study, according to the NRS and ODI scores, both groups had significant improvements in pain relief and functionality at 18 months after PELD; in terms of the MacNab criteria, 90.9% in group A and 93.2% in group B showed excellent or good outcomes; two patients in group A and 3 patients in group B exhibited additional lumbar surgery. We found that PELD is an effective surgical procedure for the treatment of LDH in adolescents, regardless of LSTV status. Lee et al. followed 46 adolescents for a mean duration of 37.2 months; 91.3% of the patients showed excellent or good outcomes, and 1 patient had a recurrence (2.2%) [3]. Chen et al. reported on 19 adolescent patients with a follow-up duration of 3–5 years: 1 patient (5.3%) exhibited a recurrence, no major complications occurred, and the success rate was 100% at the last follow-up [6]. Our findings correspond to those reported in the literature.

More importantly, the mean NRS scores for LBP or leg pain, ODI score, and MacNab grade at 18 months after surgery between the 2 groups were not significantly different, respectively. Therefore, LSTV did not affect LBP or leg pain relief or life function improvement in the short term after PELD. These results contradict our hypothesis. We speculated that there are three possible reasons: (1) the intervertebral disc in adolescents is usually hydrated, rubbery, and viscous [3], which may predispose them to LDH recurrence [6]. Residual nucleus pulpoda tissue and the surrounding inflammatory factors stimulate the nerve root, which may be the main reason for recurrent back pain or leg pain, while LSTV has a relatively small effect on back pain or leg pain; (2) LSTV is associated with LDH and LBP in adolescents [19, 21]. However, in the short term after surgery, LSTV might have little effect on low back pain or disc herniation. Perhaps a longer follow-up will yield different results; (3) LSTV is mainly associated with LBP but not leg pain. The LBP is relatively mild and can be alleviated with conservative treatment and has less impact on pain intensity and life function than leg pain. Therefore, the influence of LSTV on poor postoperative outcomes is relatively small.

Two patients (one patient had a recurrence; one patient had a new lumbar disc herniation) in group A and 3 patients (one patient had a recurrence; two patients had new lumbar disc herniations) in group B underwent additional surgery, and all patients achieved a good recovery at the follow-up time. There was no significant difference between the 2 groups in the incidence of additional lumbar surgery in the short term after PELD. Imaging analysis showed that patients undergoing additional surgery for a new segmental disc herniation often had multisegmental lumbar disc herniation on MRI before the first operation (Figure 4). Our study found that a new lumbar disc herniation is an important reason for additional surgery in adolescents.

Adolescents with lumbar disc herniation and LSTV are a special group with unique imaging characteristics. The sacralization of L5 may protect the L5/S1 level and contribute to the L4/5 disc herniation [10, 11, 19, 21, 22]. However, we found that many adolescents with LSTV also had L5/S1 disc herniation. Many adolescents in both groups had multisegmental lumbar disc herniation on MRI (22.7% in group A; 43.2% in group B), which may be prone to new lumbar disc herniation after the first operation. Patients with LSTV have a larger SSA and a higher iliac crest. This finding is in accordance with some previous studies [23, 24]. Since the high iliac crest increases the difficulty of posterolateral puncture, we needed to adjust the puncture angle or remove some of the ventral bone of the superior facet.

Few studies have evaluated the efficacy of lumbar discectomy in adolescent patients with LSTV. Ahn et al. reported that 31 patients with LSTV and 35 patients without LSTV were followed up for at least 2 years after microdiscectomy (MD); LSTV can limit a patient's clinical improvement after MD regarding pain intensity and recurrence [25]. Their surgical procedures and the study population were different from our study, which may affect the results.

This study has several limitations: (1) it was a retrospective study with a small sample size (22 vs. 44); (2) there was only one follow-up point, which could not reflect the trend of pain relief and life function improvement postoperatively; (3) the follow-up time was short (18 months); and (4) the surgeries were performed by 2 different surgeons. Our research also has advantages: (1) to reduce the risk of confounding factors, we prepared homogeneous populations in 2 groups regarding age, sex, BMI, surgeons, and physical activity; we excluded patients if they had any other spine diseases, if they were operated on by any other doctors, or if their operation level was not at the L4/5 or L5/S1 single level and (2) the patient's clinical and imaging data were sufficient; we associated the imaging data with clinical outcomes for analysis. However, multicenter, large-sample, randomized, controlled, and prospective studies are necessary to confirm our findings.

## 5. Conclusion

Our study suggests that in terms of pain relief, life function improvement, and the incidence of additional lumbar surgery, LSTV has no effect on the short-term clinical outcomes of PELD in adolescents. A new lumbar disc herniation is an important reason for additional surgery in adolescents, regardless of their LSTV status.

## Figures and Tables

**Table 1:** Comparison of demographic characteristics.

Characteristics	Group A (*n* = 22)	Group B (*n* = 44)	*p* value
Age (years)	19.5 (17.8 ~ 20.3)	20.0 (18.3 ~ 21.0)	0.391^∗^
Sex, n			1.000*^+^*
Male	18	36	
Female	4	8	
BMI (kg/m^2^)	25.1 ± 4.2	24.6 ± 3.9	0.641^#^
Ratio of two surgeons, *n*	20/2	36/8	0.544*^+^*
Duration of symptoms (months)	6.0 (2.8 ~ 12.0)	6.0 (4.0 ~ 12.0)	0.428^∗^
History of injury, *n*	5	7	0.498*^+^*
Clinical symptoms, *n*			0.150*^+^*
Hip pain	2	0	
LBP	0	1	
Leg pain	2	8	
Pain in the back and leg	18	35	
Positive straight leg raise test, *n*	19	40	0.888*^+^*

Values are presented as mean ± SD, Md (*P*25 ~ *P*75), or number. BMI: body mass index; LBP: low back pain. ^∗^Mann–Whitney *U* test. *^+^*Pearson's chi-square test or Fisher's exact test. ^#^*t*-test.

**Table 2:** Comparison of imaging characteristics.

Characteristics	Group A (*n* = 22)	Group B (*n* = 44)	*p* value
Operated level, *n* (%)			0.473*^+^*
L4/5	15 (68.2)	26 (59.1)	
L5/S1	7 (31.8)	18 (40.9)	
Type of herniation, *n* (%)			0.702*^+^*
Central	11 (50)	19 (43.2)	
Centrolateral	11 (50)	24 (54.5)	
Foraminal	0	1 (2.3)	
Herniation level on MRI, *n* (%)			0.332*^+^*
L4/5	10 (45.5)	11 (25.0)	
L5/S1	7 (31.8)	14 (31.8)	
Multisegment	5 (22.7)	19 (43.2)	
L4/5, L5/S1	3	12	
L3/4, L4/5	0	1	
L3/4, L4/5, L5/S1	1	5	
L2/3, L3/4, L4/5	1	0	
L2/3, L4/5, L5/S1	0	1	
Calcification, *n* (%)	5 (22.7)	10 (22.7)	1.000*^+^*
SSA (degrees)	33.5 (26.8 ~ 36.3)	25.0 (21.0 ~ 29.8)	<0.001^∗^
LLA (degrees)	28.0 (17.5 ~ 40.5)	29.0 (20.0 ~ 32.8)	0.395^∗^
Iliac crest height (mm)	15.6 (12.6 ~ 24.6)	0.0 (-1.9 ~ 6.9)	<0.001^∗^

Values are presented as Md (*P*25 ~ *P*75), or number (%). SSA: sacral slope angle; LLA: lumbar lordosis angle. ^∗^Mann–Whitney *U* test. *^+^*Pearson's chi-square test or Fisher's exact test.

**Table 3:** Clinical outcome evaluation.

Variable	Group A (*n* = 22)	Group B (*n* = 44)	*p* value
NRS, lower back pain			
Preoperative	4.0 (3.0 ~ 4.0)	3.0 (3.0 ~ 4.0)	0.081^∗^
18 months postop	0.5 (0.0 ~ 2.0)	0.0 (0.0 ~ 2.0)	0.764^∗^
NRS, leg pain			
Preoperative	5.0 (5.0 ~ 6.0)	6.0 (5.0 ~ 6.0)	0.156^∗^
18 months postop	0.0 (0.0 ~ 0.0)	0.0 (0.0 ~ 0.0)	0.213^∗^
ODI			
Preoperative	34.2 (28.4 ~ 39.6)	34.2 (28.8 ~ 37.8)	0.874^∗^
18 months postop	7.2 (3.6 ~ 16.2)	12.6 (5.9 ~ 16.2)	0.117^∗^
MacNab grading system, *n* (%)			1.000*^+^*
Excellent/good	12/8 (90.9)	22/19 (93.2)	
Fair/poor	0/2 (9.1)	0/3 (6.8)	
Additional lumbar surgery, *n* (%)	2 (9.1)	3 (6.8)	1.000*^+^*
Recurrence	1	1	
A new lumbar disc herniation	1	2	
Postoperative pain occurred, *n* (%)			0.541*^+^*
Hip pain	0	2 (4.5)	
LBP	8 (36.4)	13 (29.5)	
Leg pain	0	0	
Pain in back and leg	4 (18.2)	6 (13.6)	

Values are presented as Md (*P*25 ~ *P*75), or number (%). NRS: numerical rating scale; ODI: Oswestry Disability Index. ^∗^Mann–Whitney *U* test. *^+^*Pearson's chi-square test or Fisher's exact test.

## Data Availability

The data used to support the findings of this study are available from the corresponding author upon request.
